# Immunoglobulin GM (γ marker) and FcγR genotypes interact to contribute to the magnitude of ADCC against SARS CoV-2 S-transfected cells

**DOI:** 10.1007/s00251-025-01385-9

**Published:** 2025-09-01

**Authors:** Janardan P. Pandey, Aryan M. Namboodiri, Paul J. Nietert

**Affiliations:** 1 Department of Pharmacology and Immunology, Medical University of South Carolina, Charleston, SC 29425 USA; 2 Department of Public Health Sciences, Medical University of South Carolina, Charleston, SC USA

**Keywords:** GM allotypes, FcγRIIIa, ADCC, SARS CoV-2, Humoral immunity

## Abstract

Immunoglobulin GM (γ marker) and KM (κ marker) allotypes have been shown to be associated with antibody responses to several viruses, but their role in immunity to severe acute respiratory syndrome coronavirus-2 (SARS-CoV-2)—the causative agent of Coronavirus disease 2019 (COVID-19)—has not been investigated. The aim of the present investigation was to determine the contribution of GM, KM, and FcγR genotypes to the magnitude of humoral immunity to SARS-CoV-2 and to the antibody-dependent cell-mediated cytotoxicity (ADCC) of SARS CoV-2 S-transfected cells. ADCC is a major host immunosurveillance mechanism against viruses and the leading mechanism underlying the clinical efficacy of therapeutic monoclonal antibodies. We genotyped 124 unvaccinated people for several GM, KM, and FcγR alleles, measured IgG antibodies to the receptor-binding domain of the spike protein (S-RBD) of SARS CoV-2, and quantitated the level of ADCC against SARS CoV-2 S-transfected cells. None of the associations between genotypes and antibody levels were statistically significant, potentially a reflection of relatively small sample sizes. However, we found a significant interactive effect of GM and FcγRIIIa valine (V)/phenylalanine (F) genotypes on the ADCC of SARS CoV-2 S-transfected cells. In the FcγRIIIa F/F group, the mean ADCC value was significantly (p = 0.03) lower among those with GM 17/17 (mean = 45.2) when compared to those with GM 3/3 (mean = 60.2). In the FcγRIIIa V/V group, the mean ADCC value was not significantly (p = 0.68) lower among those with GM 17/17 (mean = 52.5) when compared to those with GM 3/3 (mean = 55.4). These results may help devise potent immunotherapy against emerging SARS CoV-2 variants.

Coronavirus disease 2019 (COVID-19)—caused by severe acute respiratory syndrome coronavirus-2 (SARS-CoV-2)—has resulted in several million deaths worldwide. Although mRNA vaccines elicit high titers of spike-specific antibodies and offer high level of protection against SARS-CoV-2 infection, new variants of the virus are steadily emerging, reducing the efficacy of the vaccine-generated antibodies. Therefore, continued investigations aimed at broadening our understanding of the immunobiology of the virus are essential. The antigen-binding fragment (Fab) of the anti-SARS-CoV-2 antibodies—generated by infection or vaccination—directly binds the antigen and provides protection through neutralization of the virus. The crystallizable fragment (Fc) of the antibody affects the effector functions, such as antibody-dependent cell-mediated cytotoxicity (ADCC). Growing body of evidence suggests an important role for the Fc-mediated effector functions in the outcome of SARS-CoV-2 infection (Zhang et al. 2023).

IgG antibody mediated ADCC is triggered upon ligation of FcγR to the Fc region of IgG molecules. It follows that genetic variation in FcγR and Fc—where virtually all GM alleles are expressed—could contribute to the differences in the magnitude of ADCC. Immunoglobulin GM (γ marker) and KM (κ marker) allotypes have been shown to be associated with immunity to several viruses, such as HCV, HIV, CMV, EBV (Pandey et al. 2008, 2014; Deepe et al. 2012; Biggar et al. 1984), but their roles in immunity to SARS-CoV-2 have not been determined. Thus, the aim of the present investigation was to determine the contribution of various GM, KM, and FcγR genotypes to the magnitude of humoral immunity to SARS-CoV-2 and to the ADCC of SARS CoV-2 S-transfected cells.

The study population comprised 124 unvaccinated people who screened positive for COVID-19 during the Medical University of South Carolina’s screening program in 2020. The study was approved by the IRB for human research. IgG antibodies to the receptor-binding domain of the spike protein (S-RBD) of SARS CoV-2 were measured by a standard ELISA method, using His Tag Recombinant SARS-CoV-2 Spike Protein (RBD: aa319-541, Thermo Fisher Scientific, Waltham, MA) as antigen. Samples with high titers of anti-S-RBD antibodies were used for affinity purification of GM 3 or GM 17-expressing IgG1 antibodies to be used in the ADCC assays. Antibodies of IgG2, IgG3 and IgG4 subclasses were removed from the total IgG by affinity elimination using monoclonal anti-human IgG2, IgG3 and IgG4 antibody-immobilized matrix. IgG1 concentration was measured by a quantitative ELISA assay using monoclonal anti-IgG for immobilization and polyclonal anti-human IgG HRP conjugate for detection, using monoclonal antibody cetuximab as a standard. Six serum samples, each homozygous for GM 3 or 17, were used in ADCC analysis.

IgG1 allotypes GM 3 and 17 were determined by a previously described TaqMan® genotyping assay (Pandey et al. 2021). The κ chain determinants KM 1 and 3 were characterized by a previously described polymerase chain reaction-restriction fragment length polymorphism (PCR-RFLP) technique (Moxley and Gibbs 1992). Genotyping of FcγRIIa histidine (H)/arginine (R) and FcγRIIIa valine (V) and phenylalanine (F) alleles was performed by RT-PCR, using pre-designed TaqMan® genotyping assays from Applied Biosystems Inc.

293-SARS2-S-dfur cells—generated from the human embryonic kidney (HEK)−293 cell line by transfection of the original Wuhan-Hu-1 SARS-CoV-2 Spike (D614) gene (InvivoGen San Diego, CA)—were used as targets in the ADCC assays. NK effector cells were isolated from blood samples homozygous for FcγRIIIa V or F alleles by affinity depletion of non-NK cells, using a kit from Milteneyi Biotec (Auburn, CA, USA), according to the manufacturer's protocol.

ADCC was measured by assaying the lactate dehydrogenase (LDH) activity. Briefly, HEK 293-SARS2-S-dfur target cells were coated with affinity purified anti-S-RBD antibodies (500 ng/ml) expressing particular GM allotype in RPMI-1640 medium containing 1% IgG free human serum and incubated for 30 min at 37 C in a carbon dioxide incubator. NK effector cells expressing FcγRIIIa V or F allele were added at a target to effector ratio of 1:10. Contents were spun at 500 RPM to bring the cells in contact and incubated for 4 hrs. Cells were spun at 1500 RPM, and the spent medium was collected and assayed for LDH activity, using the Cytotox-96 kit from Promega Corporation (Madison, WI, USA). Spontaneous LDH release, due possibly to killer-cell immunoglobulin-like receptor dependent cytotoxicity, from target cells incubated with NK cells without anti-S-RBD antibody, was used as blank (negative control). Total LDH content, in the target cells incubated with NK cells, was calculated by the lysis of the cells in 1% triton X100 (positive control). Results are expressed as percentage of LDH release in test wells compared to triton X100 lysed cells, after subtracting the absorbance values obtained for blank at 495 nm.

$$\mathrm{ADCC}\left(\%\right)=\frac{\text{LDH activity in the test well}-\text{LDH activity in the blank}}{\text{LDH activity in the lysed cells}-\text{LDH activity in the blank}}\times 100$$

Affinity purified anti-S-RBD IgG1 antibodies from six COVID-19 patients expressing the GM 17 allotype and six patients expressing the GM 3 allotype were employed in the ADCC assays. ADCC experiments involving each combination of GM (3/17) and FcγRIIIa (V/F) were replicated six times.

Anti-S-RBD IgG antibody levels were not normally distributed. Therefore, a series of non-parametric Wilcoxon rank sum tests were conducted to compare the antibody levels associated with and without the minor alleles. For comparison of the ADCC values associated with different combinations of GM (3/3, 17/17) and FcγRIIIa (V/V, FF) genotypes, a mixed linear regression model was used. This model included a random subject effect with a compound symmetry covariance structure to account for the intraclass correlation among individual subject’s six repeated measurements. For all analyses, p<0.05 was considered statistically significant. No corrections for multiple comparisons were made, due to the hypothesis generating nature of the study. For such exploratory studies, it has been suggested that data be analyzed without multiplicity adjustment (Bender and Lange 2001).

Table 1 presents the demographics of the study population and anti-S-RBD IgG antibody levels, stratified by genotypes. None of the associations between genotypes and antibody levels were statistically significant, potentially a reflection of relatively small sample sizes. For example, when the analyses were not stratified by race, subjects with GM 23 +/+ or GM 23 +/- (median: 105.9 AU/µl) had significantly (p=0.01) higher antibody levels when compared to those with GM 23 -/- (median: 78.5 AU/µl).

**Table 1:** Anti-S-RBD IgG antibodies (AU/μl), stratified by race and genotype

Genotype	N	Median concentration	25th percentile	75th percentile	Wilcoxon Rank Sum Test P-Value
**Whites**	88				
Males/Females	23/65				
Mean age (SEM)	47.9 (12.0)				
KM 1/1 or KM 1/3	24	91.3	35.2	492.5	0.52
KM 3/3	59	97.0	66.3	355.3	
GM 6 + / + or +/-	0				
GM 6 -/-	81	91.0	49.3	216.8	
GM 17/17 or GM 3/17	47	117.8	47.8	449.0	0.41
GM 3/3	37	91.8	50.0	202.5	
GM 23 + / + or GM 23+/-	57	103.3	68.3	422.5	0.13
GM 23 -/-	30	79.0	31.0	194.8	
FCGR3A V/V or V/F	49	100.8	66.3	354.8	0.42
FCGR3A F/F	39	91.0	46.5	364.3	
FCGR2A H/H or H/R	58	85.9	49.3	354.8	0.52
FCGR2A R/R	27	185.3	69.8	422.5	
**Blacks**	36				
Males/Females	17/19				
Mean age (SEM)	50.5 (3.16)				
KM 1/1 or KM 1/3	28	56.3	35.8	180.9	0.20
KM 3/3	8	124.9	73.4	288.9	
GM 6 + / + or +/-	16	121.8	44.7	206.1	0.13
GM 6 -/-	19	42.0	32.8	157.3	
GM 17/17 or GM 3/17	33	84.0	37.5	187.0	0.73
GM 3/3	3	73.3	35.5	748.5	
GM 23 + / + or GM 23 +/-	3	668.3	44.8	748.5	0.09
GM23 -/-	32	78.7	36.8	180.9	
FCGR3A V/V or V/F	16	103.4	37.8	199.4	0.56
FCGR3A F/F	20	65.9	30.8	172.2	
FCGR2A H/H or H/R	25	73.3	36.0	187.0	0.68
FCGR2A R/R	11	84.0	42.0	187.8	

Table 2 presents the levels of anti-S-RBD IgG antibody-mediated ADCC (%) of SARS CoV-2 S-transfected cells by NK cells, stratified by GM and FcγRIIIa genotypes. Results of the mixed linear regression analyses of the ADCC data are as follows. In the FcγRIIIa F/F group, the mean ADCC value was significantly (p=0.03) lower among those with GM 17/17 (mean=45.2, SE=3.0) when compared to those with GM 3/3 (mean=60.2, SE=5.3). In the FcγRIIIa V/V group, the mean ADCC value was not significantly (p=0.68) lower among those with GM 17/17 (mean=52.5, SE=1.7) when compared to those with GM 3/3 (mean=55.4, SE=6.8).

**Table 2:** Anti-S-RBD IgG antibody-mediated ADCC (%) of SARS CoV-2 S-transfected cells by NK cells, stratified by GM and FcγRIIIa genotypes

NK cell genotype	GM Genotype	Mean	SE	p-Value
FcγRIIIA F/F	GM 3/3	60.2	5.3	0.03
FcγRIIIA F/F	GM 17/17	45.2	3.0	
FcγRIIIA V/V	GM 3/3	55.4	6.8	0.68
FcγRIIIA V/V	GM 17/17	52.5	1.7	

The most interesting finding in the present investigation is that GM 3-expressing anti-S-RBD IgG1 antibodies interact with phenylalanine-expressing FcγRIIIa expressed on NK cells and induce higher ADCC of SARS CoV-2 S-transfected cells than GM 17-expressing antibodies. Affinity usually correlates with toxicity. It is possible that GM 3-expressing IgG1 antibodies have higher affinity to phenylalanine-expressing FcγRIIIa. This appears to be consistent with the results of an IgG-FcγRIIIa binding study, in which the authors reported that for both FcγRIIIa valine and phenylalanine expressing NK cells, GM 3-expresing IgG1 proteins bound slightly (but reproducibly) better than those expressing the GM 1,17 allotypes (Armour et al. 2010). We did not type for the GM 1 allotype in this investigation, but all GM 17 positive people are most likely positive for GM 1 as well, as the two alleles are in absolute linkage disequilibrium (Lefranc and Lefranc 2012)

These results provide a mechanistic explanation for the GM allotype associated severity of COVID-19 reported earlier (Vázquez-Coto et al. 2024). In a study of Spanish COVID-19 patients who needed treatment in the intensive care unit, we found that GM 17-carrying patients were at almost three-fold higher risk of death than non-carriers, and that GM 3 was associated with a protective effect. The higher death rate among GM 17-expressing patients could, at least in part, be due to the reduced potency of their anti-S-RBD IgG1 antibodies to induce ADCC of SARS CoV-2. In the previous study we did not type the patients for FcγRIIIa alleles, but a majority of them were probably positive for the phenylalanine variant, since it is the major allele.

GM allotypes have been shown to be associated with ADCC against two other viruses—CMV and HSV1 (Vietzen et al. 2016; Moraru et al. 2015). The present report is the first documenting their role in the ADCC of SARS CoV-2. It needs to be replicated in an independent investigation. We did not find a significant association between GM, KM, and FcγR alleles and anti-S-RBD antibodies in this investigation. A larger study population is needed to conclusively determine the putative role of these genes in humoral immunity to SARS CoV-2.

This study has certain limitations. The study cohort was relatively small (124 individuals), which reduces the statistical power. Also, the subjects were from a single institution and divided in two racial groups—blacks and white. Since there are racial differences in gene frequencies, the findings reported here may not be generalizable to other racial groups.

## Data Availability

No datasets were generated or analysed during the current study.
